# The Sick and the Hospitals

**Published:** 1887-06-04

**Authors:** Andrew Clark, Henry C. Burdett

**Affiliations:** President; Chairman of Executive Committee


### The Sick and the Hospitals.

The following scheme, approved by the Council of The Hospitals Association, Norfolk House, Norfolk Street, London, W.C., cannot fail to be of interest to the Clergy and Ministers of religion, as well as to Boards of Guardians, District Visitors, Hospital Officials and Governors, and others:—

One of the objects of The Hospitals Association is to afford precise information relative to the resources of the London and Provincial Hospitals; since it is admitted that delays, dangerous to health and life, are sometimes experienced in placing deserving cases in suitable institutions.

To carry out this object, and also the other objects relating to the organisation, working, and maintenance of hospitals, for which the Association was founded, it is necessary to secure the co-operation and association of all persons interested in the treatment of the sick poor, and especially of the managers of Hospitals, Boards of Guardians, the Clergy and Ministers of all denominations, and local representative Societies.

We therefore earnestly solicit your consideration of the following scheme of affiliation adopted by the Council of The Hospitals Association, which, if successful, will bring into communication all persons interested in dealing with the sick poor. This scheme will facilitate and enlarge the means of helping them, and will bring within the reach of every member of the Association an authentic and accurate knowledge of the economy and work of Hospitals and of their claims to public support.

I. All persons interested in the treatment of the sick poor, especially the managers of Hospitals, Boards of Guardians, the Clergy and Ministers of all denominations, and local representative Societies, are invited to affiliate themselves to The Hospitals Association in London by the formation of a Local Hospital Association, of which at least one local Medical Practitioner should be a Member.

II. The Hospitals Association will be the central and representative organisation, and its office in London will be a Bureau of Information, a Medium of Communication, and a Centre of Reference. Its aim will be to supply all kinds of information on Hospital questions; to give advice respecting the organisation, management, and maintenance of all classes of Hospitals; to arrange for lectures to such Local Hospital Associations as may desire them; and to render assistance in the management of public meetings for the furtherance of Hospital purposes.

III. Every affiliated Local Hospital Association may nominate a representative, who will enjoy all the privileges of an individual Member of the Association, and will be eligible for election to any of its honorary offices.

IV. The inclusive annual subscription for each Member, or of each affiliated Local Association, will be one guinea per annum.

V. The Journal of the Association, The Hospital, which is published weekly, will be sent post free to each Member of the Association, and to every representative of an affiliated Local Association.

In committing this scheme to your favourable consideration, we desire to renew the expression of our conviction that affiliation to The Hospitals Association will greatly promote the interests of the sick poor, give much additional help to all who are concerned in caring for them, and contribute to the better understanding, and the more liberal support of the great work carried on by our Hospitals in the healing of the sick, the advancement of medical knowledge, and the dissemination of the experience so acquired for the common service of mankind.

We enclose a form of application for Membership of The Hospitals Association in the case of individuals, and for affiliation in the case of Local Hospital Associations.

Any further information may be obtained on application to the Secretary of The Hospitals Association, Norfolk House, Norfolk Street, Strand, London, W.C.

We are, Sir,
Your faithful servants,
ANDREW CLARK, M.D.,
*President.*
HENRY C. BURDETT,
*Chairman of Executive Committee.*

### A Year's Work in the Hospitals and Medical Charities of London.

The following Tables and Figures should be studied with the remarks following the Summary on the last page, to make their bearing more intelligible and better understood.

### NEWINGTON AND SOUTH DISTRICT.

Comprising Battersea, Wandsworth, Tooting, Balham, Clapham, Streatham, Brixton, Lambeth, Newington, Southwark, Bermondsey, Camberwell, Greenwich, Deptford, Lewisham, Blackheath, Woolwich, Sydenham, etc.

| No. of Beds. | No. of Beds daily occupied. | Hospitals. | In-patients. | Out-patients. | Total Expenditure. | Income. | | | Total Income. |
|---|---|---|---|---|---|---|---|---|---|
| | | | | | | Charitable. | Proprietary. | Patients' Payments | |
| | | | | | £ | £ | £ | £ | £ |
| 24 | 18 | Miller ... ... ... ... ... | 175 | 14,727 | 3,239 | 2,1[illegible]2 | 281 | ...... | 2,463 |
| 258 | 180 | Seamen's ... ... ... ... | 2,267 | 5,359 | 12,256 | 5,969 | 4,693 | 127 | 10,789 |
| 60 | 57 | Evelina ... ... ... ... | 511 | 10,427 | 5,149 | 3,813 | 804 | 105 | 4,722 |
| 45 | 39 | Home for Sick Children... ... | 255 | 1,161 | 1,409 | 1,189 | 3 | 255 | 1,447 |
| 24 | 18 | General Lying-in... ... ... | 383 | 866 | 2,987 | 837 | 2,142 | ...... | 2,979 |
| 51 | 48 | Royal, for Women and Children | 483 | 7,942 | 2,888 | 2,101 | 687 | 200 | 2,988 |
| 14 | 8 | Royal South London Ophthalmic | 130 | 5,968 | 1,151 | 956* | 113 | ...... | 1,069 |
| 10 | 6 | Hospital for Diseases of the Skin | 31 | 5,882 | 1,138 | 419 | 99 | 653 | 1,171 |
| 500 | 221 | Metropolitan Convalescent ... | 3,359 | ...... | 6,454 | 5,671 | 379 | 63 | 6,113 |
| 986 | 595 | | 7,594 | 52,332 | 36,671 | 23,137 | 9,201 | 1,403 | 33,741 |
| | | **Dispensaries.** | | | | | | | |
| ... | ... | Battersea ... ... ... ... | ...... | 5,478 | 1,833 | 108 | 34 | 1,693 | 1,835 |
| ... | ... | Brixton, etc. ... ... ... | ...... | 3,815 | 624 | 502 | 62 | 70 | 634 |
| ... | ... | Camberwell Provident ... ... | ...... | 11,330 | 1,740 | 398 | 78 | 1,192 | 1,668 |
| ... | ... | Clapham ... ... ... ... | ...... | 1,897 | 444 | 426 | ...... | 101 | 527 |
| ... | ... | Forest Hill ... ... ... | ...... | 3,592 | 576 | 261 | ...... | 326 | 587 |
| ... | ... | Gipsy Hill... ... ... ... | ...... | 2,145 | 291 | 66 | ...... | 227 | 293 |
| ... | ... | Royal South London ... ... | ...... | 5,491 | 652 | 689 | 99 | ...... | 788 |
| ... | ... | South Lambeth ... ... ... | ...... | 2,214 | 595 | 445 | 5 | 206 | 656 |
| ... | ... | South London Medical Aid ... | ...... | 1,517 | 505 | 90 | 42 | 260 | 392 |
| ... | ... | Wandsworth Common ... ... | ...... | 1,798 | 200 | 73 | ...... | 109 | 182 |
| ... | ... | West Dulwich ... ... ... | ...... | 416 | 59 | 17 | ...... | 29 | 46 |
| 986 | 595 | | 7,594 | 92,025 | 44,190 | 26,212 | 9,521 | 5,616 | 41,349 |

* Also a legacy of £27,000 Consols.

### CITY AND EAST CENTRAL DISTRICT.

Comprising the City, St. Luke's, Finsbury, and Clerkenwell.

| No. of Beds. | No. of Beds. daily occupied. | Hospitals. | In-patients. | Out-patients. | Total Expenditure. | Income. | | | Total Income. |
|---|---|---|---|---|---|---|---|---|---|
| | | | | | | Charitable. | Proprietary. | Patients' Payments. | |
| | | | | | £ | £ | £ | £ | £ |
| 160 | Nil. | Metropolitan Free ... ... | ...... | 27,828 | 3,522 | 3,603 | 276 | ...... | 3,879 |
| 160 | 138 | Royal Free... ... ... ... | 1,813 | 22,191 | 11,492 | 3,143 | 1,599 | ...... | 4,742 |
| 80 | 30 | Royal Hosp. for Dis. of the Chest | 305 | 5,470 | 4,060 | 2,781 | 307 | ...... | 3,088 |
| 33 | 13 | City of London Lying-in ... | 283 | 1,249 | 2,926 | 302 | 2,455 | 10 | 2,767 |
| 34 | 23 | St. Mark's ... ... ... ... | 322 | 939 | 2,221 | 1,133 | 813 | ...... | 1,946 |
| 100 | 92 | Royal London Ophthalmic ... | 2,049 | 26,211 | 6,154 | 2,295 | 1,050 | ...... | 3,345 |
| 25 | 20 | City Orthopædic ... ... ... | 134 | 3,045 | 1,327 | 1,580 | 70 | ...... | 1,650 |
| 592 | 316 | | 4,906 | 86,933 | 31,702 | 14,837 | 6,570 | 10 | 21,417 |
| | | **Dispensaries.** | | | | | | | |
| ... | ... | City ... ... ... ... ... | ...... | 4,179 | 1,339 | 929 | 189 | ...... | 1,118 |
| ... | ... | City of London and East London | ...... | 5,739 | 632 | 203 | 38 | 633 | 874 |
| ... | ... | Farringdon ... ... ... | ...... | 8,292 | 809 | 262 | ...... | 389 | 651 |
| ... | ... | Finsbury ... ... ... ... | ...... | 15,912 | 797 | 652 | 112 | 283 | 1,047 |
| ... | ... | Metropolitan ... ... ... | ...... | 5,415 | 658 | 353 | 120 | 200 | 673 |
| ... | ... | Royal General ... ... ... | ...... | 3,684 | 858 | 734 | 233 | 92 | 1,059 |
| ... | ... | Royal Maternity Charity ... | ...... | 4,000 | 3,164 | 943 | 958 | ...... | 1,901 |
| 592 | 316 | | 4,906 | 134,154 | 39,959 | 18,913 | 8,220 | 1,607 | 28,740 |

### WESTMINSTER DISTRICT.

Comprising Westminster City and Liberties.

| No. of Beds. | No. of Beds daily occupied. | Hospitals. | In-patients. | Out-patients. | Total Expenditure. | Income. | | | Total Income. |
|---|---|---|---|---|---|---|---|---|---|
| | | | | | | Charitable. | Proprietary. | Patients' Payments. | |
| | | | | | £ | £ | £ | £ | £ |
| 154 | 115 | Charing Cross ... ... ... | 1,618 | 23,101 | 13,407 | 6,127 | 3,020 | ...... | 9,147 |
| 206 | 159 | King's College ... ... ... | 2,048 | 16,459 | 17,031 | 12,248 | 1,772 | 54 | 14,074 |
| 200 | 142 | Westminster ... ... ... | 1,864 | 15,058 | 17,870 | 6,282 | 3,035 | ...... | 9,317 |
| 14 | 11 | Grosvenor, for Women & Children | 87 | 2,112 | 1,084 | 622 | 14 | 367 | 1,003 |
| 50 | 20 | Royal Westminster Ophthalmic | 319 | 7,112 | 1,780 | 1,452 | 274 | 91 | 1,817 |
| 50 | 45 | Royal Orthopædic ... ... | 134 | 1,132 | 2,021 | 1,309 | 317 | 138 | 1,764 |
| 18 | 10 | Hospital for Diseases of Throat | 182 | 4,332 | 2,693 | 651 | 79 | 1,868 | 2,598 |
| 10 | 10 | West End, Nervous System ... | 44 | 623 | 1,223 | 931 | 4 | 442 | 1,377 |
| 114 | 106 | Ventnor, for Consumption ... | 637 | ...... | 16,565 | 5,249 | 2,032 | 2,421 | 9,702 |
| 816 | 618 | | 6,933 | 69,929 | 73,674 | 34,871 | 10,547 | 5,381 | 50,799 |
| | | Dispensaries. | | | | | | | |
| ... | ... | Public ... ... ... ... | ...... | 3,748 | 691 | 753 | 171 | ...... | 924 |
| ... | ... | SS. George and James ... ... | ...... | 3,001 | 748 | 412 | 12 | ...... | 424 |
| ... | ... | St. George, Hanover Square ... | .. ... | 2,465 | 706 | 499 | ...... | 191 | 690 |
| ... | ... | Western ... ... ... ... | ...... | 3,690 | 939 | 410 | 261 | 279 | 950 |
| ... | ... | Westminster, General ... ... | ...... | 5,604 | 561 | 287 | 145 | 37 | 469 |
| 816 | 618 | | 6,933 | 88,417 | 77,319 | 37,232 | 11,136 | 5,888 | 54,256 |

### ST. MARYLEBONE AND WEST CENTRAL DISTRICT.

Comprising St. Marylebone, St. John's Wood, Bloomsbury, Holborn, etc.

| No. of Beds. | No. of Beds daily occupied. | Hospitals. | In-patients. | Out-patients. | Total Expenditure. | Income. | | | Total Income. |
|---|---|---|---|---|---|---|---|---|---|
| | | | | | | Charitable. | Proprietary. | Patients' Payments. | |
| | | | | | £ | £ | £ | £ | £ |
| 36 | 25 | French ... ... ... ... | 387 | 2,826 | 2,746 | 3,499 | 219 | ...... | 3,718 |
| 51 | 47 | SS. John and Elizabeth ... ... | 82 | ...... | 1,544 | 916 | 625 | 26 | 1,567 |
| 310 | 267 | The Middlesex ... ... ... | 2,856 | 35,933 | 23,197 | 8,878 | 5,229 | ...... | 14,107 |
| 80 | 70 | Alexandra, for Children... ... | 145 | 195 | 2,272 | 1,586 | 89 | 682 | 2,357 |
| 181 | 132 | Hospital for Sick Children ... | 1,358 | 15,066 | 10,568 | 11,753 | 675 | ...... | 12,428 |
| 56 | 32 | Queen Charlotte's Lying-in ... | 885 | 1,106 | 3,805 | 2,133 | 530 | 38 | 2,701 |
| 125 | 99 | Hosp. for Paralysed and Epileptic | 519 | 3,042 | 8,786 | 4,580 | 1,527 | 1,450 | 7,557 |
| 13 | 7 | Central London Ophthalmic ... | 159 | 7,518 | 921 | 369 | 77 | 196 | 642 |
| 20 | 6 | Western Ophthalmic ... ... | 72 | 2,035 | 718 | 376 | 104 | 179 | 659 |
| 26 | 20 | New Hospital for Women ... | 223 | 3,079 | 2,009 | 1,623 | 192 | 497 | 2,312 |
| 38 | 37 | National Orthopædic ... ... | 108 | 751 | 1,072 | 518 | ...... | 720 | 1,238 |
| 25 | 19 | St. John's for Diseases of Skin ... | 164 | 4,298 | 3,227 | 2,494 | 44 | 758 | 3,296 |
| 17 | 14 | Central London Throat and Ear | 315 | 5,434 | 1,830 | 883 | 36 | 970 | 1,889 |
| 80 | 57 | London Homœopathic ... ... | 675 | 8,844 | 4,685 | 2,618 | 1,638 | 367 | 4,623 |
| ... | ... | Dental ... ... ... ... | ...... | 29,645 | 1,394 | 1,458 | 78 | 177 | 1,713 |
| 22 | 8 | British Lying-in ... ... ... | 162 | 753 | 1,845 | 456 | 995 | 20 | 1,471 |
| 52 | 49 | Samaritan Free ... ... ... | 538 | 4,648 | 5,336 | 4,027 | 97 | ...... | 4,124 |
| 20 | 12 | Italian ... ... ... ... | 169 | 2,092 | 606 | 498 | 21 | ...... | 519 |
| 30 | 29 | Home for Incurable Children ... | 42 | ...... | 1,258 | 899 | 17 | 455 | 1,371 |
| 25 | 17 | Establishment for Gentlewomen | 121 | ...... | 2,378 | 1,159 | 86 | 1,003 | 2,248 |
| 1,207 | 947 | | 8,980 | 127,265 | 80,197 | 50,723 | 12,279 | 7,538 | 70,540 |
| | | Dispensaries. | | | | | | | |
| ... | ... | Bloomsbury ... ... ... | ...... | 3,637 | 803 | 283 | 60 | ...... | 348 |
| ... | ... | Margaret Street Infirmary ... | ...... | 3,000 | 425 | 352 | 154 | ...... | 506 |
| ... | ... | Portland Town ... ... ... | ...... | 1,793 | 154 | 151 | ...... | 16 | 167 |
| ... | ... | Portobello Road ... ... ... | ...... | 283 | 59 | 31 | ...... | 34 | 65 |
| ... | ... | St. John's Wood ... ... ... | ...... | 1,634 | 539 | 263 | 14 | 290 | 567 |
| ... | ... | St. Marylebone, General ... ... | ...... | 3,994 | 831 | 388 | 127 | 274 | 789 |
| ... | ... | Western, General ... ... ... | ...... | 17,349 | 1,443 | 1,247 | 50 | ...... | 1,29[illegible] |
| 1,207 | 947 | | 8,980 | 158,955 | 84,451 | 53,438 | 12.684 | 8,152 | 74,274 |

### KENSINGTON AND WEST DISTRICT.

Comprising Kensington, Paddington, Bayswater, Kilburn, Chelsea, Brompton, Fulham, Hammersmith, Chiswick, Brentford, Acton, Ealing, etc.

| No. of Beds. | No. of Beds daily occupied. | Hospitals. | In-patients. | Out-patients. | Total Expenditure. | Income. | | | Total Income. |
|---|---|---|---|---|---|---|---|---|---|
| | | | | | | Charitable. | Proprietary. | Patients' Payments. | |
| | | | | | £ | £ | £ | £ | £ |
| 351 | 269 | St. George's ... ... ... | 3,357 | 22,959 | 26,805 | 11,198 | 8,063 | ...... | 19,261 |
| 281 | 203 | St. Mary's ... ... ... ... | 2,352 | 22,443 | 19,925 | 12,693 | 1,319 | ...... | 14,012 |
| 101 | 33 | West London ... ... ... | 591 | 10,004 | 4,568 | 4,607 | 58 | ...... | 4,665 |
| 337 | 297 | Hospital for Consumption ... | 1,610 | 14,050 | 23,950 | 14,412 | 3,657 | ...... | 18,069 |
| 22 | 15 | Belgrave, for Children ... ... | 116 | 2,969 | 1,305 | 1,164 | 10 | 2 | 1,176 |
| 27 | 20 | Paddington Green, do. ... ... | 309 | 11,338 | 2,078 | 3,095 | 78 | 171 | 3,344 |
| 74 | 70 | Victoria, do. ... ... | 729 | 9,433 | 5,261 | 3,900 | 532 | 293 | 4,725 |
| 63 | 40 | Chelsea, for Women ... ... | 490 | 3,274 | 3,173 | 3,562 | 49 | 813 | 4,424 |
| 188 | 80 | Female Lock ... ... ... | 461 | ...... | 4,038 | 2,519 | ...... | 1,469 | 3,988 |
| 20 | 11 | Male Lock ... ... ... ... | 203 | 2,458 | 1,180 | 332 | ...... | 731 | 1,063 |
| 105 | 76 | Cancer ... ... ... ... | 652 | 976 | 8,536 | 6,073 | 800 | ...... | 6,873 |
| 66 | 50 | Hospital for Women ... ... | 587 | 5,129 | 6,258 | 4,781 | 103 | 1,565 | 6,449 |
| 25 | 19 | National, for Diseases of Heart | 90 | 2,601 | 2,182 | 1,696 | 84 | 366 | 2,146 |
| ... | ... | National Dental ... ... ... | ...... | 22,122 | 651 | 411 | ...... | 392 | 803 |
| 24 | 24 | Cheyne, for Incurable Children | 43 | ...... | 1,383 | 1,374 | 231 | 222 | 1,827 |
| 1,684 | 1,207 | | 11,590 | 129,756 | 111,293 | 71,817 | 14,984 | 6,024 | 92,825 |
| | | **Dispensaries.** | | | | | | | |
| ... | ... | Brompton, Provident ... ... | ...... | 819 | 390 | 177 | 4 | 237 | 418 |
| ... | ... | Chelsea, Brompton, etc. ... | ...... | 5,274 | 488 | 374 | 113 | ...... | 487 |
| ... | ... | Kensington ... ... ... | ...... | 4,978 | 789 | 778 | 42 | ...... | 820 |
| ... | ... | Kilburn, Maida Vale ... ... | ...... | 3,618 | 548 | 470 | 44 | ...... | 514 |
| ... | ... | Kilburn, Provident ... ... | ...... | 4,414 | 1,023 | 97 | 99 | 828 | 1,024 |
| ... | ... | Notting Hill, Provident... ... | ...... | 4,171 | 276 | 194 | 25 | 93 | 312 |
| ... | ... | Paddington, Provident ... ... | ...... | 8,753 | 628 | 215 | 22 | 416 | 653 |
| ... | ... | Royal Pimlico ... ... ... | ...... | 6,289 | 827 | 306 | 6 | 546 | 858 |
| ... | ... | SS. Paul and Barnabas ... ... | ...... | 2,613 | 508 | 377 | ...... | 127 | 504 |
| ... | ... | Westbourne, Provident ... ... | ...... | 3,136 | 383 | 110 | 8 | 264 | 382 |
| 1,684 | 1,207 | | 11,590 | 173,821 | 117,153 | 74,915 | 15,347 | 8,535 | 98,797 |

### ISLINGTON AND NORTH AND NORTH-WEST DISTRICT.

Comprising Islington, Holloway, Highbury, Hampstead, St. Pancras, Tottenham, Kentish Town, etc.

| No. of Beds. | No. of Beds daily occupied. | Hospitals. | In-patients. | Out-patients. | Total Expenditure. | Income. | | | Total Income. |
|---|---|---|---|---|---|---|---|---|---|
| | | | | | | Charitable. | Proprietary. | Patients' Payments. | |
| | | | | | £ | £ | £ | £ | £ |
| 33 | 30 | Great Northern Central... ... | 279 | 8,787 | 4,025 | 2,518 | 285 | ... | 2,803 |
| 46 | 41 | North West London ... ... | 602 | 10,637 | 3,289 | 3,083 | 40 | 117 | 3,240 |
| 50 | 40 | Tottenham... ... ... ... | 552 | 5,079 | 3,038 | 2,625 | 369 | 153 | 3,147 |
| 209 | 184 | University College ... ... | 2,836 | 37,377 | 20,599 | 10,523 | 4,821 | 73 | 15,417 |
| 38 | 35 | North London Consumption ... | 316 | 2,700 | 4,236 | 4,156 | 80 | 34 | 4,270 |
| 36 | 12 | St. Saviour's, for Cancer ... | 86 | 274 | 3,356 | 277 | 76 | 901 | 1,254 |
| 190 | 47 | London Fever ... ... ... | 582 | ...... | 7,599 | 5,727 | 664 | 2,302 | 8,693 |
| 25 | 17 | Hospital for Epilepsy, etc. ... | 107 | 757 | 2,101 | 1,481 | ... | 606 | 2,087 |
| 67 | 52 | London Temperance ... ... | 624 | 3,252 | 4,011 | 4,125 | 297 | 333 | 4,755 |
| 16 | 8 | Hampstead Home Hospital ... | 91 | ...... | 1,360 | 767 | 187 | 405 | 1,359 |
| 28 | 17 | Invalid Asylum ... ... ... | 168 | ...... | 891 | 557 | 72 | 210 | 839 |
| 738 | 483 | | 6,243 | 68,863 | 54,505 | 35,839 | 6,891 | 5,134 | 47,864 |
| | | **Dispensaries.** | | | | | | | |
| ... | ... | Hampstead Provident ... ... | ...... | 9,066 | 846 | 320 | 10 | 530 | 860 |
| ... | ... | Holloway and North Islington... | ...... | 5,969 | 1,072 | 674 | 9 | 447 | 1,130 |
| ... | ... | Islington ... ... .. ... | ...... | 13,983 | 2,426 | 279 | 558 | 474 | 1,311 |
| ... | ... | St. Pancras and Northern ... | ...... | 2,236 | 526 | 307 | 111 | 73 | 491 |
| ... | ... | Child's Hill ... ... ... | ...... | 517 | 294 | 48 | 17 | 235 | 300 |
| ... | ... | Stamford Hill ... ... ... | ...... | 5,215 | 677 | 527 | 98 | ... | 625 |
| 738 | 483 | | 6,243 | 105,849 | 60,346 | 37,994 | 7,694 | 6,893 | 52,581 |

### STRATFORD AND EAST-END DISTRICT.

Comprising Bethnal Green, Tower Hamlets, Stratford, West Ham, Whitechapel, Hackney, Stepney, Limehouse, Poplar, and the East.

| No. of Beds. | No. of Beds daily occupied. | HOSPITALS. | In-patients. | Out-patients. | Total Expenditure. | Income. | | | Total. Income. |
|---|---|---|---|---|---|---|---|---|---|
| | | | | | | Charitable. | Proprietary. | Patients' Payments. | |
| | | | | | £ | £ | £ | £ | £ |
| 790 | 629 | London ... ... ... ... | 8,102 | 80,591 | 70,003 | 24,898 | 18,117 | 139 | 43,154 |
| 142 | 123 | German ... ... ... ... | 1,861 | 22,003 | 9,187 | 7,393 | 2,167 | 286 | 9,852 |
| 50 | 36 | Poplar ... ... ... ... | 625 | 8,738 | 3,070 | 3,807 | 312 | ...... | 4,119 |
| 164 | 105 | City of London Chest ... ... | 794 | 15,662 | 11,255 | 7,025 | 343 | ...... | 7,368 |
| 92 | 71 | East London Children ... ... | 950 | 14,460 | 6,850 | 5,377 | 410 | ...... | 5,787 |
| 64 | 51 | North-Eastern do. ... ... | 711 | 14,083 | 4,349 | 2,854 | 292 | 740 | 3,886 |
| 91 | 78 | Mrs. Gladstone's Convalescent ... | 1,153 | ...... | 1,595 | 894 | 369 | ...... | 1,263 |
| 1,393 | 1,093 | | 14,196 | 155,537 | 106,309 | 52,248 | 22,016 | 1,165 | 75,429 |
| | | DISPENSARIES. | | | | | | | |
| ... | ... | Eastern ... ... ... ... | ...... | 5,153 | 667 | 188 | 249 | 50 | 487 |
| ... | ... | London ... ... ... ... | ...... | 3,267 | 447 | 181 | 270 | ...... | 451 |
| ... | ... | Queen Adelaide's ... ... ... | ...... | 4,274 | 616 | 530 | 130 | ...... | 660 |
| ... | ... | Tower Hamlets ... ... .. | ...... | 4,007 | 512 | 326 | 28 | 139 | 493 |
| ... | ... | West Ham, Stratford .. ... | ...... | 17,163 | 848 | 916 | 129 | ...... | 1,045 |
| 1,393 | 1,093 | | 14,196 | 189,401 | 109,399 | 54,389 | 22,822 | 1,354 | 78,565 |

### SUMMARY.

It will be seen from the following Summary that the Hospitals and Medical Charities of London during the twelve months ending 31st December, 1886, relieved upwards of 1,003,000 patients, at a total cost of £532,000. The ordinary income from all sources was only a little over £428,500, so that there was an actual deficiency on the year's working of upwards of £100,000.

| No. of Beds. | No. of Beds daily occupied. | HOSPITALS. | In-patients. | Out-patients. | Total Expenditure. | Income. | | | Total Income. |
|---|---|---|---|---|---|---|---|---|---|
| | | | | | | Charitable. | Proprietary. | Patients' Payments. | |
| | | | | | £ | £ | £ | £ | £ |
| 986 | 595 | Newington and South District... | 7,594 | 92,025 | 44,190 | 26,212 | 9,521 | 5,616 | 41,349 |
| 592 | 316 | City and East Central do. ... | 4,906 | 134,154 | 39,959 | 18,913 | 8,220 | 1,607 | 28,740 |
| 816 | 618 | Westminster District ... ... | 6,933 | 88,417 | 77,319 | 37,232 | 11,136 | 5,888 | 54,256 |
| 1,207 | 947 | St. Marylebone & West Central do. | 8,980 | 158,955 | 84,451 | 53,438 | 12,684 | 8,152 | 74,274 |
| 1,684 | 1,207 | Kensington and West District... | 11,590 | 173,821 | 117,153 | 74,915 | 15,347 | 8,535 | 98,797 |
| 736 | 483 | Islington & North-West District | 6,243 | 105,849 | 60,346 | 37,994 | 7,694 | 6,893 | 52,581 |
| 1,393 | 1,093 | Stratford and East End do. | 14,196 | 189,401 | 109,399 | 54,389 | 22,822 | 1,354 | 78,565 |
| 7,414 | 5,259 | | 60,442 | 942,622 | 532,817 | 303,093 | 87,424 | 38,045 | 428,562 |

### AN APPEAL ON THE FACTS.

OUT of four million inhabitants it will be seen that upwards of one million *directly* received relief from the hospitals during this year alone. Who shall estimate the amount of benefit which the hospitals have *indirectly* given to Londoners in addition, through the medical profession and skilled nurses, whom the hospitals have trained to minister to those who can afford to be treated at their own homes in the hour of sickness? Towards defraying the cost to the hospitals of directly relieving in a single year this grand total of human misery, the charitable and proprietary revenue, with the patients' payments contributed, were inadequate to the extent of £100,000, a deficiency which would have been half as much again, except for the money at present subscribed on Hospital Sunday. We ask the wealthy, the thoughtful, and the generous to study the above figures. They will show to all how each charity does its work, and what is the principle upon which that work is carried on.

Some people hold, with Mr. Spurgeon, that the managers of a charity should never be waiters upon Providence, but that funds should be collected and placed in the bank before orders are given for the work to be undertaken. This list will be found to contain institutions which have managed to make both ends meet and a little over, so that Mr. Spurgeon's principles are well represented. Unfortunately, however, it is not always possible to conduct a hospital on such business-like principles, or many sick and suffering folk would fare badly indeed at times. To be of the greatest utility, a hospital has often to be placed in the midst of a dense and indigent population, who have not the means of supporting it adequately, and the managers have therefore to appeal to the charitable who live in the wealthier parts of the town. In such circumstances it necessarily happens that the hospital is in a chronic state of impecuniosity, and many such institutions will be found with the others given here. It is not too much to say that the very varying condition of the hospitals of London revealed in these Tables must include every conceivable variety of circumstance, and so be calculated to meet the views of every group of philanthropists. This fact, coupled with the remembrance that the crusade to be conducted throughout the Hospitals Week, as a prologue to Hospital Sunday, is a united movement in favour of all the hospitals, should call forth an amount of enthusiasm worthy of so great a cause, and the pressing pecuniary needs of a class of charities the efficient maintenance of which is a matter of vital importance to all classes of the community. At least £100,000 is needed. Shall it not be forthcoming? Will the flowers of so fragrant a garden be neglected by men and women who have means? The receipts on Hospital Sunday will afford the best answer. In London to-day

> "The charities which soothe, and heal, and bless,
> Lie scattered at the feet of men like flowers."

Who will not gather a button-hole at least, if a bouquet be indeed beyond their resources?

